# Dr. W. G. Rockwood

**Published:** 1909-05-01

**Authors:** 


The death is announced from Ceylon of Dr. W. G. Rockwood, who was very generally regarded as the leading practitioner of the island. We learn from the *Times* that he was born at Jaffna in 1843, and was educated in India, receiving his professional training at the Madras Medical College. Thence he returned to Ceylon and entered the Government service, occupying the post of medical officer at Puttalam from 1867 to 1875. He obtained the M.D. degree of the Madras University, passing in the first class with special honours, and receiving exceptional commendation from the Examining Board. Dr. Rockwood held many important medical appointments during his professional career in Ceylon. He was for many years surgeon to the Colombo General Hospital (subsequently being elected consulting surgeon) and lecturer in surgery and midwifery at the Ceylon Medical College. For ten years he was a member of the Ceylon Legislative Council, and was honoured by his colleagues in the island by the presidency of the Ceylon branch of the British Medical Association. He frequently visited Europe, and when in England on leave he obtained the diplomas of M.R.C.S. and M.R.C.P., and in 1902 he represented the local branch of the British Medical Association at the annual meeting at Manchester. He retired from active work three years ago owing to failing health.

